# A complete high-quality MinION nanopore assembly of an extensively drug-resistant *Mycobacterium tuberculosis* Beijing lineage strain identifies novel variation in repetitive PE/PPE gene regions

**DOI:** 10.1099/mgen.0.000188

**Published:** 2018-06-15

**Authors:** Arnold Bainomugisa, Tania Duarte, Evelyn Lavu, Sushil Pandey, Chris Coulter, Ben J. Marais, Lachlan M. Coin

**Affiliations:** ^1^​ Institute for Molecular Bioscience, The University of Queensland, Brisbane, Australia; ^2^​ Faculty of Medicine, The University of Queensland, Brisbane, Australia; ^3^​ Central Public Health Laboratory, Port Moresby, Papua New Guinea; ^4^​ Queensland Mycobacteria Reference Laboratory, Brisbane, Australia; ^5^​ Marie Bashir Institute for Infectious Diseases and Biosecurity, University of Sydney, Sydney, Australia

**Keywords:** *Mycobacterium tuberculosis*, Oxford Nanopore Technologies

## Abstract

A better understanding of the genomic changes that facilitate the emergence and spread of drug-resistant *Mycobacterium tuberculosis* strains is currently required. Here, we report the use of the MinION nanopore sequencer (Oxford Nanopore Technologies) to sequence and assemble an extensively drug-resistant (XDR) isolate, which is part of a modern Beijing sub-lineage strain, prevalent in Western Province, Papua New Guinea. Using 238-fold coverage obtained from a single flow-cell, *de novo* assembly of nanopore reads resulted into one contiguous assembly with 99.92 % assembly accuracy. Incorporation of complementary short read sequences (Illumina) as part of consensus error correction resulted in a 4 404 064 bp genome with 99.98 % assembly accuracy. This assembly had an average nucleotide identity of 99.7 % relative to the reference genome, H37Rv. We assembled nearly all GC-rich repetitive PE/PPE family genes (166/168) and identified variants within these genes. With an estimated genotypic error rate of 5.3 % from MinION data, we demonstrated identification of variants to include the conventional drug resistance mutations, and those that contribute to the resistance phenotype (efflux pumps/transporter) and virulence. Reference-based alignment of the assembly allowed detection of deletions and insertions. MinION sequencing provided a fully annotated assembly of a transmissible XDR strain from an endemic setting and showed its utility to provide further understanding of genomic processes within *Mycobacterium tuberculosis*.

## Data Summary

1. Sample Illumina and MinION sequencing reads generated and analysed are available in NCBI under project accession number PRJNA386696 (https://www.ncbi.nlm.nih.gov/sra/?term= PRJNA386696).

2. The assembled complete genome and its annotations are available in NCBI under accession number CP022704.2 (https://www.ncbi.nlm.nih.gov/sra/?term=CP022704.2).

**Impact Statement**

We recently characterized a modern Beijing lineage strain of *Mycobacterium tuberculosis* responsible for the drug resistance outbreaks in Western Province, Papua New Guinea. As some of the genomic markers responsible for drug resistance and transmissibility among this strain remain unknown, there is a need to elucidate all molecular mechanisms that account for the resistance phenotype, virulence and transmission. Whole genome sequencing using short reads has been widely utilized to study the *M. tuberculosis* genome but it does not generally capture long repetitive regions as variants in these regions are eliminated during analysis. Illumina instruments are known to have a GC bias so that regions that are GC- or AT-rich are under-sampled and this effect is exacerbated in *Mycobacterium tuberculosis*, which has approximately 65 % GC content. In this study, we utilized Oxford Nanopore Technologies (ONT) MinION sequencing to assemble a high-quality complete genome of an extensively drug-resistant strain of a modern Beijing lineage. We were able to able to assemble all PE/PPE (proline-glutamate/proline-proline-glutamate) gene families that are GC-rich and repetitive in nature. We show the utility of ONT in offering a more comprehensive understanding of the genetic mechanisms that contribute to resistance, virulence and transmission. This is important for setting up predictive analytics platforms and services to support diagnostics and treatment in endemic settings with drug-resistant tuberculosis.

## Introduction

Globally, the tuberculosis (TB) incidence rate has shown a slow decline over the last two decades, although absolute case numbers continue to rise due to population growth, with an estimated 10.4 million new cases occurring in 2016 [1]. TB control gains are threatened by the growing number of drug-resistant strains recorded around the world [2]. An estimated 490 000 incident cases of multi-drug-resistant (MDR) TB, i.e. resistance to at least isoniazid and rifampicin, occurred in 2016 [1]. The incidence of extensively drug-resistant (XDR) strains, i.e. MDR strains with additional resistance to at least one fluoroquinolone and second-line injectable, is also on the rise [1]. Further multiplication of drug resistance in strains that are already highly drug-resistant could lead to programmatically incurable TB, where construction of a curative regimen might be impossible with existing treatment options [3, 4]. Management of drug-resistant TB places a major financial burden on health systems, which may be overwhelmed in settings with high disease burdens [3].

In the absence of lateral gene transfer [5], drug resistance in *Mycobacterium tuberculosis* (MTB) arises mainly from chromosomal mutations that are selected by chemotherapeutic pressure, which drives drug resistance multiplication and the ongoing evolution of drug-resistant strains [6–8]. Successful transmission of drug-resistant strains results in clonal expansion and potential epidemic spread [9–11]. The acquisition of resistance-conferring mutations has potential to lead to epidemic spread if these drug-resistant strains are readily transmissible [12, 13]. The mechanisms underlying the development of highly transmissible XDR strains are not fully elucidated. One such mechanism is the induction of efflux pumps, which may lead to high-level resistance in mycobacteria [14], without any metabolic compromise. While previous studies described efflux pump genes and identified mutations in some of these genes [15, 16], efflux pumps in a transmissible XDR strain have not been described.

Whole genome sequencing using short reads has elucidated a large number of mutations associated with drug resistance, as well as compensatory mutations, but has limited capacity to resolve large structural variations, gene duplications or variations in repetitive regions [10, 17, 18]. Long-read sequencing could provide a more comprehensive understanding of the evolutionary mechanisms underlying the emergence of highly transmissible drug-resistant strains [17]. In principle, Oxford Nanopore Technologies MinION sequencing technology offers read lengths that are only limited by the length of DNA presented and produces data in real time [19]. The small size, ease of use and cheap unit cost of the MinION nanopore sequencer facilitates successful deployment in resource-limited settings, as has been achieved during the Ebola outbreak in West Africa [20]. Although the potential of MinION to detect drug resistance mutations in *M. tuberculosis* has been demonstrated [21], its application for complete MTB genome assembly has not been reported.

Papua New Guinea (PNG) has a high rate of drug-resistant TB in its Western Province [22]. We recently characterized a drug-resistant TB outbreak on Daru island, which is driven by a modern Beijing sub-lineage 2.2.1.1 strain [23]. Whilst some genetic markers within the strain have been identified [23], the molecular mechanisms responsible for pathogenesis and virulence are not fully elucidated. Genomic regions such as proline-glutamate (PE, 99 loci) and proline-proline-glutamate (PPE, 69) genes are thought to contribute to virulence and pathogenicity of a strain [24, 25] and yet they are routinely excluded from data analysis after whole genome sequencing [9, 23, 26]. These gene families constitute signature motifs near the N terminus of their gene products and are subclassified according to sequence features on the C terminus. PE genes are divided into PE_PGRS (polymorphic GC-rich sequence, 65 genes) and PE (no distinctive feature, 34 genes) while PPE genes are divided into PPE_MPTR (major polymorphic tandem repeats, 23 genes), PPE_SVP (Gxx-SVPxxW motif, 24 genes), PPE_PPW (PxxPxxW motif, 10 genes) and PPE (no distinctive feature, 12 genes) [24]. The existence of these subgroups highlights the diversity in their roles towards immune invasion and virulence [27].

Because of the repetitive nature of nucleotides and GC-rich content within the PE/PPE gene regions, they are a source of sequencing error from short-read sequencing such as Illumina. Previous studies have utilized Illumina sequencing to provide understanding of the role of these gene families but some of the reported results are variable and inconsistent with no complete assembly of these genes [27–29]. A study by Elghraoui and colleagues utilized small molecule real-time (SMRT) sequencing, which produces long reads, to assemble a complete genome of MTB H37Ra that included all the PE/PPE gene families [30]. However, this was performed on an attenuated strain, and there remains a need to use cheap long-read sequencing technology on drug-resistant strains to provide insight into the underlying mechanisms of drug resistance and identify key features associated with virulence and transmissibility. We utilized MinION to assemble a comprehensive genome of an XDR strain including variable and repetitive regions such as PE/PPE genes. The assembly will serve as an ideal reference for ongoing MDR/XDR outbreak surveillance in Western Province, PNG, and the far north of Queensland.

## Methods

### Phenotypic susceptibility testing

We selected an XDR isolate originating from the Western Province of PNG, which we refer to as the WP-XDR strain. This strain was tested for resistance to first- and second-line drugs including: rifampicin (1.0 µg ml^−1^), isoniazid (0.1 µg ml^−1^, low-level; and 0.4 µg ml^−1^, high-level), streptomycin (1.0 µg ml^−1^), ethambutol (5.0 µg ml^−1^), pyrazinamide (100 µg ml^−1^) and second-line drugs amikacin (1.0 µg ml^−1^), capreomycin (2.5 µg ml^−1^), kanamycin (2.5 µg ml^−1^), ethionamide (5.0 µg ml^−1^), ofloxacin (2.0 µg ml^−1^), *p*-aminosalicylic acid (4.0 µg ml^−1^) and cycloserine (50 µg ml^−1^). The automated Bactec Mycobacterial Growth Indicator Tube (MGIT) 960 system (Becton Dickinson) was used for susceptibility testing offirst- and second-line drugs. A minimal inhibitory concentration assay for second-line drugs was performed using the Sensititre (TREK diagnostic system) system.

### DNA extraction and quantification

DNA was extracted from Lowenstien–Jensen (LJ) slopes cultured for 4 weeks at 37 °C. DNA isolation was performed using mechanical and chemical methods. Briefly, five glass beads (0.7 mm; Sigma) were added to 200 µl of PrepMan Ultra sample preparation reagent (Thermo Fisher Scientific). Full loops of culture were added to the reagent and mixed well. The solution was dry heat incubated for 10 min at 95 °C, followed by bead beating for 40 s at 6.0 m s^−1^ using a mini-beadbeater-16 (BioSpec Products). It was then centrifuged for 10 min at 13^ ^000 r.p.m. before transferring 40 µl of the supernatant to another vial. We added 45 µl of 3 M sodium acetate and 1 ml of ice-cold ethanol (96 %), centrifuged the solution at maximum speed for 15 min and removed the supernatant. We then added 1 ml of 70 % ethanol, left it at room temperature for 1 min, and again removed all the supernatant. The remaining pellet was dried for 15 min and re-suspended with 40 µl of nuclease-free water. The DNA was quantified using a Nanodrop 8000 and Qubit dsDNA HS assay kit (both from Thermo Fisher Scientific).

### MinION library preparation and sequencing

DNA was purified using 0.4× Agencourt AMPure XP beads (Beckman Coulter) and fragment distribution size was assessed using an Agilent 4200TapeStation (Agilent). Preparation of a one-dimensional genomic DNA library was performed using the SQK-LSK108 system (Oxford Nanopore Technologies, Oxford, UK) according to the manufacturer's instructions. We performed dA-tailing and end-repair using the NEBNext Ultra II End-repair/dA-tail module with two step incubation periods, of 20 min each. A purification step using 0.7× Agencourt AMPure XP beads (Beckman Coulter) was then performed according to the manufacturer's instructions. A ligation step was performed using New England Biolabs Blunt/ligase master mix module according to the manufacturer's instructions and the reaction was incubated at room temperature for 20 min. Adaptor-ligated DNA was purified using 0.4× Agencourt AMPure XP beads (Beckman Coulter) following the manufacturer's instructions but using Oxford Nanopore supplied buffers (adaptor bead binding and elution buffers). The library was then ready for MinION sequencing.

With the MinION MK1B device connected to the computer via a USB3, MinKNOW software (v.1.4.3) was started to perform quality control checks on pore activity and equilibrate the flow cell (FLO-MIN106, version R9.4). The library was combined with reagents supplied by Oxford Nanopore and loaded onto the flow cell following the manufacturer's instructions, choosing a 48 h sequencing procedure. Illumina data for the strain were available from our previous study [23].

### MinION and Illumina data analysis

Raw files generated by MinKNOW were base called using Albacore (v2.0) to return Oxford Nanopore Technologies (ONT) fastq files. *De novo* genome assembly was performed using Canu [31] and the assembly was improved using nanopolish (methylation aware option) [32]. The sequence depth of coverage of ONT reads versus the assembly was then assessed using SAMtools [33]. The assembly was further improved through two different error correction steps: Racon (utilizing raw ONT reads) and Pilon (utilizing Illumina reads) [34]. CheckM was used to assess the quality (completeness and heterogeneity) of the assemblies [35]. To determine the accuracy of the assemblies, Illumina reads of the study strain were first *de novo* assembled using SPAdes 3.11.1 [36]. The resultant Illumina assembly was used to determine the accuracy of ONT assemblies by using statistics from dnadiff (MUMmer version 3) [37]. The assembly with the highest quality was circularized using Circulator v1.5.1 [38], and similarity with the reference genome H37Rv (NC_000962.3) was calculated using nucmer (MUMmer version 3) [37] and an average nucleotide identity (ANI) calculator [39]. Genome annotation was performed using the NCBI pipeline [40] and circular representation of the genome was viewed using Circos [41]. To evaluate the assembly of PE/PPE gene families, all the PE/PPE gene sequences within MTB H37Rv were searched from Mycobrowser [42] and used to create a customized PE/PPE database. A blastn search of the assembly against the database was performed. Percentage breadth of coverage and read depth for PE/PPE gene families from ONT and Illumina reads was assessed using DepthOfCoverage (GATK version 3.7) [43] after mapping to H37Rv using BWA-MEM [44]. The resultant depths were plotted using ggplot (R statistical package). Biodiff (https://www.gitlab.com/LPCDRP/biodiff) was used to call structural variants from the assembly against H37Rv as a reference and as a query. Structural variants were further evaluated by mapping Illumina raw data of lineage-representative genomes including Indo-Oceanic, East-Asian (including Beijing lineage), East-African-Indian and Euro-American lineages and H37Rv from previous studies [23, 26, 45, 46], to the assembly and reference genome, then using Sniffles [47] on the resulting BAM files. The identified insertion sequences within the assembly were assessed for GC content using the GC calculator (Biologics International). The programs blastn and Phyre2 [48] were used to query and predict protein structure of insertion sequences within the assembly.

### Genotyping comparison between MinION and Illumina data

Using the reference genome H37Rv, SNPs and small indels (<5 bp) were called from ONT reads using nanopolish [32] and annotated using SnpEff [49]. SNPs within PE/PPE genes were characterized. Polymorphisms in known drug resistance genes (including compensatory mutations) were analysed. MycoBrowser [42] was used to annotate genes that are putatively involved in virulence, efflux pumps and cell transport, and mutations within those genes were characterized. SNPs from Illumina reads were identified using a GATK workflow (Fig. S1, available in the online verson of this article) [43], and consensus SNPs between Illumina and ONT was evaluated. Non-consensus SNPs from ONT reads were considered sequencing errors provided Illumina coverage was greater than 30×. This analysis excluded SNPs in PE/PPE genes (Table S1). An analysis pipeline composed of different software to generate variants from ONT and Illumina raw files was compiled (Fig. S1).

## Results

### Analysis of MinION-derived assembly of the WP-XDR strain

**Fig. 1.**
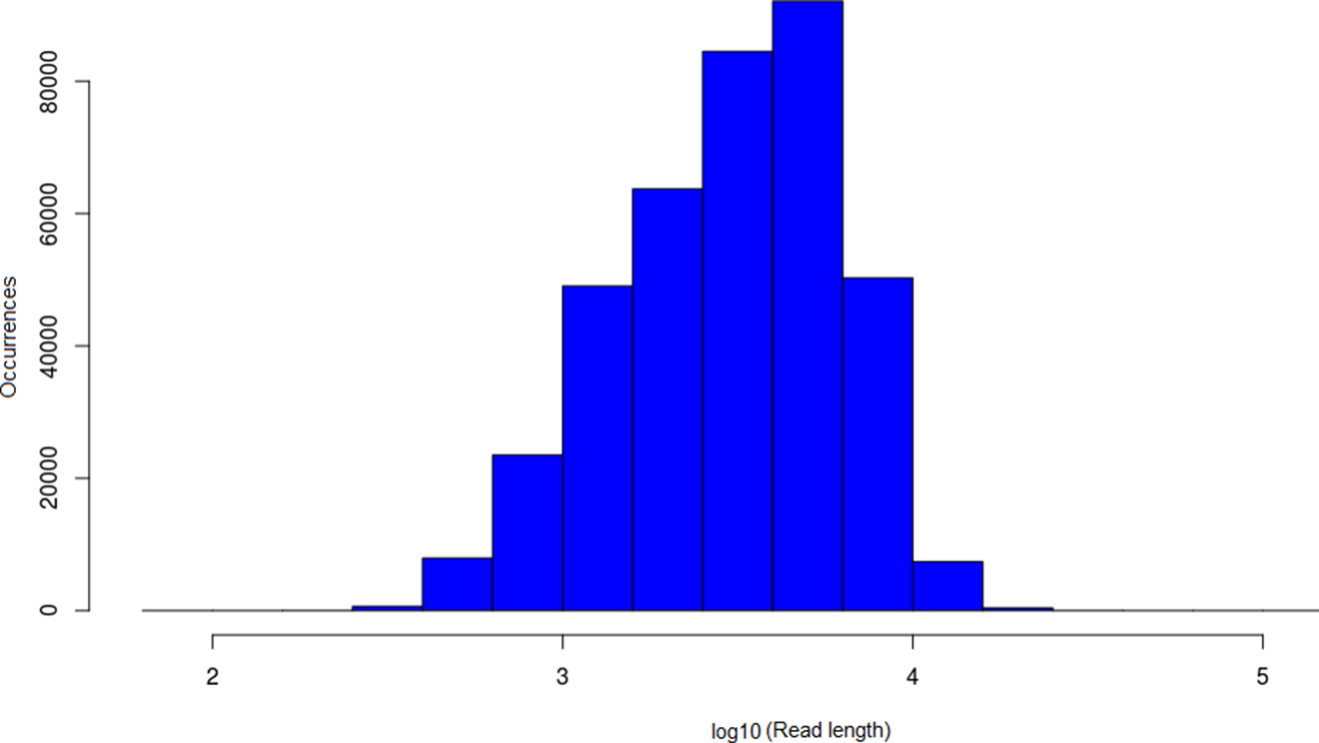
Bar graph showing the unimodal sequence length distribution from ONT.

**Fig. 2.**
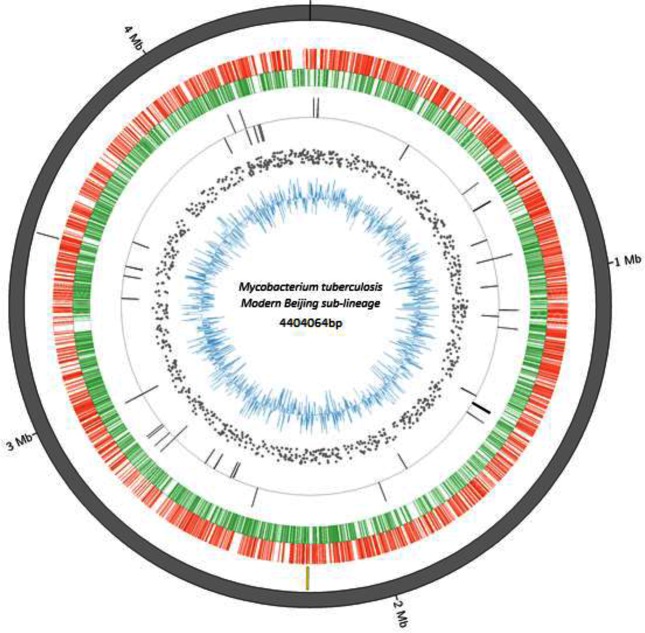
Circular representation of a WP-XDR genome (modern Beijing sub-lineage 2.2.1.1 strain) with gene annotations. From the inner to outer ring: blue, GC content of coding sequence; scattered grey, SNPs relative to H37Rv; grey bars, reverse and forward rRNA; green, reverse coding sequences; red, forward coding sequences; yellow bars, unique regions compared to H37Rv; grey outside ring, assembled contig. Mb, million base pairs.

In total, 373952 ONT reads passed base calling with N50 read length of 5073 bp, longest read 330 509 bp and average sequence depth of 238× (Fig. 1). The size of the assembled genome varied among the different consensus tools used: Racon, 4 404 947 bp; Pilon, 4 405 333 bp; and Pilon plus Racon, 4 404 064 bp. Use of raw ONT reads for error correction of the assembly resulted in a 99.92 % accurate assembly (Racon), while use of Illumina reads improved it further to 99.97 % (Pilon). According to CheckM results, the assembly from a combination of Pilon plus Racon tools had better quality scores (Table 1), and we referred to this as WP-XDR assembly. The single contig from this assembly of 4 404 064 bp had a GC content of 65.5 % and had an ANI score of 99.7 % to H37Rv (Fig. S2). NCBI annotation of the genome yielded a total of 3697 coding DNA sequences (CDS), 45 tRNAs, three rRNAs (5S, 16S, 23S) and three non-coding RNAs (Fig. 2). A blastn analysis of a PE/PPE customized database against the assembly genome showed 166/168 PE/PPE genes had >90 % nucleotide sequence similarity at an E-score ≤10^−6^ (Fig. S3). Two genes, *wag22* and PE_PGRS57, had nucleotide sequence similarity of 58.8 and 78 %, respectively. Evaluation of PE/PPE genes when ONT reads when mapped to H37Rv revealed nearly all PE/PPE genes (166/168) had 100 % breadth of coverage at an average read depth of 299.87 (IQR 285.91–311.36) (Fig. S3). Again *wag22* and PE_PGRS57 had breadth of coverage of 26 and 62 %, respectively. From Illumina reads of the study strain, only 54.3 % (92/168) of the PE/PPE genes had 100 % breadth of coverage at an average sequence depth of 46.3 (Fig. S4) with *wag22* and PE_PGRS57 having zero coverage.

**Table 1.** CheckM quality evaluation and assembly accuracy of draft genomes obtained from the three consensus tools, relative to the SPAdes assembly Racon tool used ONT reads only, Pilon used Illumina reads only and Pilon+Racon used a combination of ONT and Illumina reads.

					SPAdes assembly (4 346 566 bp)			
Tool	Genome size (bp)	Completeness (%)	Contamination	Strain heterogeneity	No. of indels	No. of SNPs	Total errors	Accuracy (%)
Racon	4 405 333	83.2	0.00	0.00	2421	1026	3447	99.92
Pilon	4 404 947	97.74	0.00	0.00	1048	236	1284	99.97
Pilon+Racon	4 404 064	100	0.00	0.00	807	217	1024	99.98

### Genotypic error analysis using MinION reads

We assessed the accuracy of MinION reads to generate accurate SNP calls in the non-repetitive part of the reference genome by excluding repetitive PE/PPE regions. A total of 1254 SNPs and 122 small indels were called from ONT reads while 1098 SNPs and 105 small indels were called from Illumina reads when mapped to H37Rv. Of these, 1095 SNPs (574 non-synonymous and 402 synonymous) and 87 small indels concurred over the technologies. In total, 118 of 159 SNPs and 23 of 35 small indels identified from ONT but not in Illumina reads were from regions of low Illumina coverage (<30× coverage). The remaining 41 SNPs (3.2 %, 41/1254) and 12 small indels (9.8 %, 12/122) were identified in ONT but missing from Illumina (high coverage regions). These might potentially be due to systematic base-calling errors that could not be corrected via consensus calling and polishing. Three (0.2 %) SNPs and 18 (14.7 %) indels were identified by Illumina but not by MinION sequencing. Considering all 194 ONT-only calls (159 SNPs and 35 indels) as false positives and 21 Illumina-only calls (three SNPs and 18 indels) as false negatives, the genotypic error rate from all ONT calls was estimated at 15.6 % [215/(1254+122)*100]. If we ignore inconsistent calls where Illumina coverage was low then the estimated genotypic error rate for base calling from ONT was reduced to 5.3 % [74/(1254+122)*100]. Counts of SNP calling among the non-consensus SNPs from ONT reads did not show any bias to a particular base (Table S2).

ONT and Illumina reads were assessed for SNP identification in PE/PPE gene families. From ONT reads, 158 SNPs were identified from 70 PE/PPE genes (42 PE and 28 PPE) with 88 SNPs (55.6 %) identified from the PE_PGRS sub-family (Table S3). From the Illumina reads, 124 SNPs from 45 PE/PPE genes (25 PE and 20 PPE) were identified, of which 31 SNPs (25 %) were from the PE_PGRS sub-family. There were 81 SNPs (from 42 PE/PPE genes) overlapping between ONT and Illumina with PPE54 having the highest number of overlapped SNPs [9] identified within one gene.

### Mutations in known resistance genes, efflux pumps and virulent genes

Phenotypic drug susceptibility results revealed the WP-XDR isolate to be extensively drug-resistant with susceptibility to only amikacin, kanamycin, *p*-aminosalicyclic acid and cycloserine. Table 2 provides details on phenotypic resistance, as well as mutations in genes known to confer drug resistance to first- and second-line drugs and recognized compensatory mutations. Genotypic drug resistance profiles concurred with phenotypic results. Ten mutations were identified in seven genes that encode trans-membrane efflux pumps and transporter proteins (Table 3). Table S4 shows 16 SNPs identified in genes that encode virulence proteins; eight (50 %) were from the *mce* gene family, and a mutation within *mycP1* (p.Thr238Ala) was also noted. In addition, 27 SNPs were identified in three genes families involved in cell wall synthesis, with 17 in *fadD*, four in *pks* and three in *mmp* gene families (Table S5).

**Table 2.** Mutations in candidate drug resistance genes identified from the WP-XDR assembly

Drug	*In vitro* phenotype	Investigated genes	Genes (mutation)
Isoniazid	Resistant	*fabG1-inhA, inhA, katG, ndh, furA, oxyR, aphC, fadE24, srmR, kasA, mshA*	*fabG1-inhA* (C-15T)
			*inhA* (p.Ile21Val)
			*ndh* (delG304)
Rifampicin	Resistant	*rpoB, rpoC, rpoA, rpoD*	*rpoB* (p.Ser450Leu)
			*rpoC* (p.Val483Gly)
Ethambutol	Resistant	*embB, embC, embA, ubiA, embR, iniA, iniC, manB*	*embB* (p.Met306Val)
Pyrazinamide	Resistant	*pncA, rpsA, panD*	*pncA* (p.Tyr103Asp)
Streptomycin	Resistant	*rpsL, gidB, rrs*	*rpsL* (p.Lys43Arg)
			*gidB* (p.Leu91Pro)
Ethionamide	Resistant	*fabG1-inhA, ethA, ethR*	*fabG1-inhA* (C-15T)
Fluoroquinolone	Resistant	*gyrA, gyrB*	*gyrA* (p.Asp94Gly)
Amikacin	Susceptible	*rrs, whib7, gidB*	Nil
Kanamycin	Susceptible	*Rv2417c-eis, whiB7, rrs, gidB*	Nil
Capreomycin	Resistant	*tlyA, whiB7, rrs, gidB*	*tlyA* (ins397C)
PAS	Susceptible	*ribD, thyA, dfrA, folC*	Nil
Cycloserine	Susceptible	*alr, ddl, cycA*	Nil

PAS, *p*-aminosalicylic acid.

**Table 3.** Mutations in putative efflux pump/transporter genes identified from the WP-XDR assembly

Genes investigated	Gene (mutation) identified	
*drrA, drrB, drrC, Rv0194, pstP,* *efpA, bacA, mmr, Rv1250,* *Rv1272c, Rv1273c, Rv1634,* *Rv1258c, mmpL13a, mmpL13b,* *P55, jefA, Rv0849, Rv2456c,* *Rv3239c, Rv2994, secA1, pstB,* *Rv2265, Rv1217, Rv1218c,* *uspA, Rv2688c, Rv1819,* *Rv1877, Rv1273c, Rv1458*	*Rv0194*	p.Met74Thr
	*Rv0194*	p.Pro1098Leu
	*secA1*	p.Asp699Glu
	*uspA*	p.Thr54Ser
	*uspA*	p.Asp67His
	*uspA*	Val127Leu
	*glnQ*	p.Met243Leu
	*Rv1218c*	p.Gln243Arg
	*Rv1250*	p.Arg278Gly
	*Rv2688c*	p.Pro156Thr

### Structural variants identified from assembly of the WP-XDR strain

**Fig. 3.**
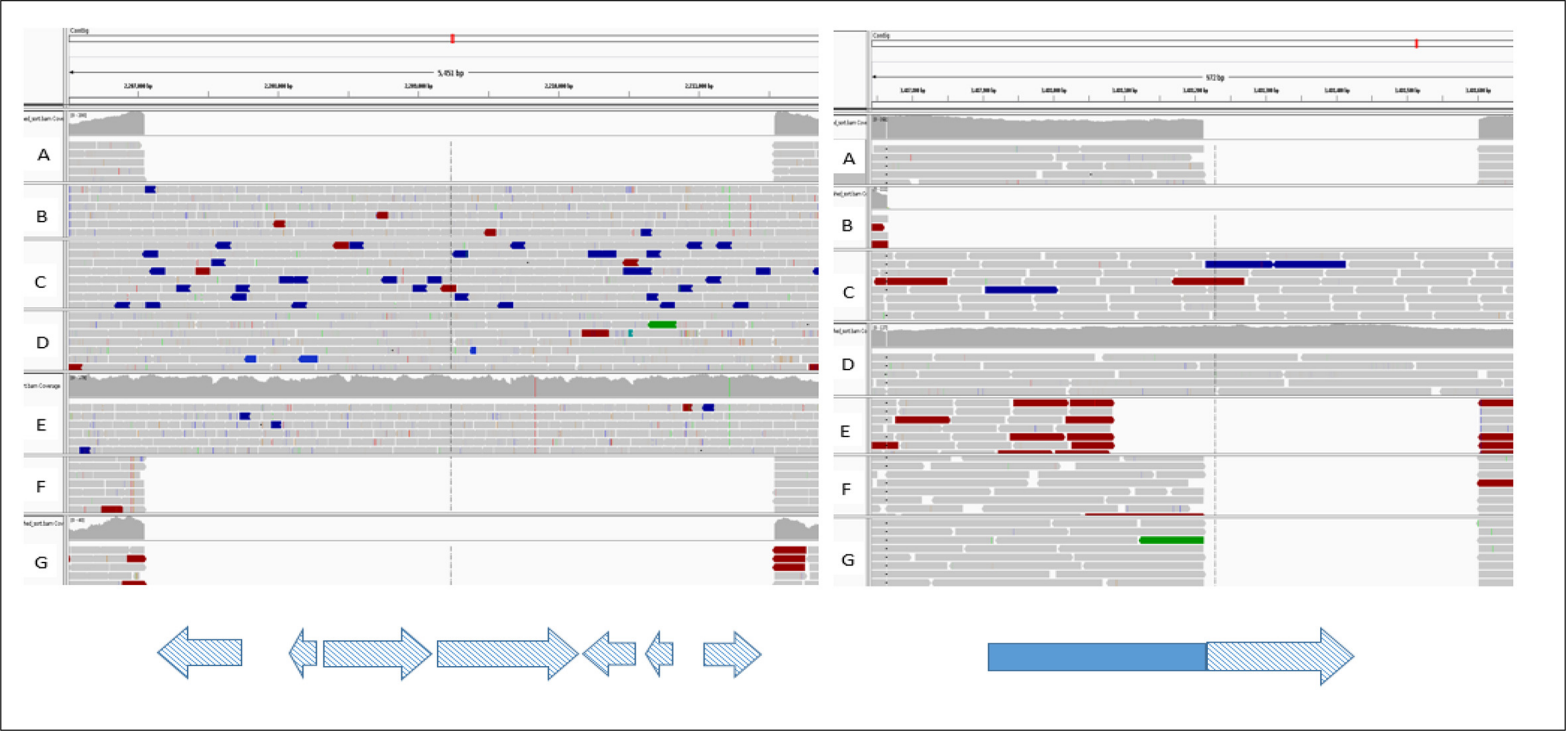
Integrative Genomic Viewer (IGV) of Illumina reads from different *M. tuberculosis* lineages (A, *M. tuberculosis* H37Rv; B, Indo-Oceanic; C, ancient Beijing; D, modern Beijing; E, East African Indian; F and G, Euro American) mapped on the ONT assembled genome highlighting the large insertions within the assembled genome. On the left: 4490 bp insertion (2 207 042–2 211 532) spanning seven annotated genes (blue checked arrows, NADH-dependent oxidoreducatse, iron-regulated elongation factor-tu, PE family protein and hypothetical/uncharacterized proteins). On the right: 390 bp insertion relative to H37Rv (3 488 211–3 488 601) spanning 323 bp (checked) at the end of a 654 bp coding sequence. ONT, Oxford Nanopore Technologies.

Two reference-based approaches were utilized using biodiff script. First, using H37Rv as a reference to the assembly, we identified known regions of difference for the Beijing sub-lineage [50] and three unknown deletions (Table 4). On evaluating the three unknown deletions using Illumina data of lineage-representative genomes mapped to the reference, Sniffles identified the three unknown deletions still missing among Beijing lineage-representative genomes. Secondly, using the assembly as a reference to H37Rv, we identified two insertions within the WP-XDR assembly of size 4490 bp (2 207 139–2 211 629) and 390 bp (3 488 211–3 488 601). On assessing Illumina data of lineage-representative genomes mapped on the WP-XDR assembly using Sniffles, the 4490 bp insertion with 64.6 % GC content was identified among Indo-Oceanic, ancient Beijing, modern Beijing and East-African-Indian lineages but absent from the Euro-American lineage (Fig. 3). As in previous studies [51–53], blastn and annotation results of the 4490 bp region was revealed to span seven complete genes that encode proteins that include NADP-dependent oxidoreductase, iron-regulated elongation factor (Tu), PE-family protein and four genes that encode uncharacterized proteins. The second smaller insertion, 390 bp with 66.7 % GC content (3 488 211–3 488 601), was identified as being unique among the Beijing lineage but varied in size as it was 835 bp with respect to Indo-Oceanic and East-African-Indian lineages (Fig. 3). Review of the NCBI annotation of the assembly indicated the insertion to be part of a 654 bp gene (CBG40_17120; 3 487 881–3 488 534) that spans 323 bp (3 488 211–3 488 534). A blast search of the insertion sequence revealed 50 % sequence identity to four *M. tuberculosis* H37Rv genomes (E-score=1.1E-175) and 100 % sequence identity to 55 *M. tuberculosis* Lineage 2 genomes (E-score=0) (Table S6). Phyre2 protein modelling [48] of the 390 bp insertion sequence predicted it to be a PPE-like protein with PE8-PPE15 protein used as the best template (79 % sequence modelled, 100 % confidence) consisting of 73 % alpha helices (Fig. S5).

**Table 4.** Characteristics of identified regions deleted from the WP-XDR assembly relative to the reference genome H37Rv

Start	Stop	Size (bp)	Region of difference	Genes deleted	RNA deleted
79 557	83 036	3479	105	Rv0071, Rv0072, Rv0073	ncRv10071, ncRv10071c
1 779 278	1 788 514	9 236	149	Rv1573, Rv1574, Rv1575, Rv1576c, Rv1577c, Rv1578c, Rv1579c, Rv1580c, Rv1581c, Rv1582c, Rv1583c, Rv1584c, Rv1585c, Rv1586c	Nil
1 986 636	1 998 623	11 987	152	plcD, Rv1756c, Rv1757c, cut1, Rv1759c, Rv1760, Rv1761c, Rv1762c, Rv1763, Rv1764, Rv1765c	Nil
2 365 414	2 366 769	1 355	Unknown*	Rv2105, Rv2106	Nil
2 535 431	2 536 142	711	181	Rv2262c, Rv2263	Nil
3 122 553	3 127 944	5 391	207	Rv2816c, Rv2817c, Rv2818c, Rv2819c, Rv2820c	Nil
3 551 229	3 552 585	1 356	Unknown*	Rv3184, Rv3185	Nil
3 552 710	3 554 025	1 315	Unknown*	Rv3186, Rv3187	Nil

*Unknown deletions in the study strain.

## Discussion

Long-read sequencing increases the potential for improved understanding of genomic factors that contribute to the *M. tuberculosis* phenotype because it has inherent power to assemble the entire strain genome. We have shown that a WP-XDR strain can be *de novo* assembled into one contig using 238× read depth data from one flow-cell of the ONT platform, reaching an accuracy of 99.92 %. As in other studies, higher genome accuracy (99.98 %) was achieved when using long reads in conjunction with Illumina reads to improve on the assembly [54, 55]. Although other long-read sequencing technologies such as SMRT have demonstrated their value in producing high-quality *M. tuberculosis* genome assemblies [30, 56], there is ongoing research aimed to improve the quality of reads produced by ONT platforms. However, ONT platforms have been shown to be cheaper and user-friendly [57], indicating its future value in TB burdened settings such as Western Province, PNG.

Our results highlighted some of the advantages of using ONT. We were able to resolve the GC-rich and highly repetitive PE/PPE gene families which were poorly resolved when Illumina reads were used. Of the two PE_PGRS subfamily genes (PE_PGRS57 and *wag22*) that had poor assembly, *wag22* has previously been shown to be deleted from Beijing lineage strains [50]. There were twice as many SNPs in PE_PGRS genes based on ONT over Illumina sequencing (Table S2). PE_PGRS gene mutations were under-represented in short-read sequencing, possibly due to their extra GC-rich motifs that could impact on sequencing. Previous studies have identified a greater number of mutations within the PE_PGRS subfamily compared to other PE subfamilies, and attribute this to their involvement in antigenic variation and immune evasion from exposure to the host immune system [27, 58]. PPE54, which is a member of a larger subfamily, PPE_MPTR (major polymorphic tandem repeat) known to have more amino-acid variations at the C-terminal site [59], had the highest number of mutations from ONT sequencing. Previous studies have shown this gene to be involved in the ‘arrest’ of phagosome maturation, allowing survival of the bacteria in the macrophages as a result of its long amino acid length at the C terminus [25, 60]. It has been postulated that PPE54 gene mutations may also play a role in development of isoniazid, rifampicin and ethambutol resistance [61], but we were unable to verify this given the presence of well-characterized drug resistance mutations.

Another advantage of ONT was the resolution of structural variants which are essential in understanding strain-related phenotypes [62]. The identification of the three deleted gene regions and the 4490 bp insertion within the assembly and other lineages but absent in H37Rv shows the limitation of H37Rv when used as a reference genome in genomic studies [51–53]. This could impact on the clear understanding of pathogenicity, virulence and transmissibility. The WP-XDR assembly has potential to be utilized as a reference genome for future investigation of XDR outbreaks in PNG. The second smaller insertion (390 bp) within the assembly had variable sizes among the reference genomes, suggesting independent structural rearrangement among the different lineages. The applied comparative approach highlights the opportunity to study lineage- and strain-specific differences, especially with the advent of long-read sequencing, which can provide complete assemblies.

Although ONT had a greater number of differing variants when compared to Illumina, it had a good estimated genotypic error rate of 5.3 %, identifying all the known drug resistance and compensatory mutations in concordance with *in vitro* susceptibility testing. This was accompanied by characterization of mutations within efflux pumps/transporter, biosynthesis pathway encoding genes (ABC, MFS, *mmpL*, *pks* and *fadD*) and virulence genes (*mce* genes*, mycp*1) which are thought contribute to the resistance phenotype and pathogenesis of the strain [63, 64]. Even with the ongoing improvement of ONT sequencing, we show that ONT data can confidently be utilized for further investigation of genomic variants that influence virulence, resistance and pathogenesis. Accompanied by the portability of ONT devices, this technology has potential for clinical utility as a point-of-care diagnostic tool because it generates reads that can be analysed in real time. This aspect was not explored in the current study but other studies have demonstrated the duration needed for speciation and identification of resistance-conferring genes [20, 65]. This is an ongoing area of research whose future application in the diagnostics of *M. tuberculosis* will greatly impact on improved management and control of infection.

Some of the limitations of the study included the use of one isolate to identify various structural variants and mutations which could be ‘background’ variations that may not impact on the strain phenotype. Use of a polished consensus assembly for analysis of structural variants could have affected detection of assembly breaks although a polishing step improved the quality of the assembly. Another limitation was the failure to confirm the presence of the identified structural variants using targeted sequencing, although the variants were consistent with Illumina data that had a high Phred score (>90).

In conclusion, we report the value of a portable small sequencer that can sequence an XDR strain, assemble a complete genome including GC-rich repetitive regions and analyse variants with high certainty. Future application of this technology in high-burden settings will have positive impacts on TB control and case management.

## Data bibliography

1. NCBI project accession number PRJNA386696 (2018).
2. GenBank accession numbers AP018034 (HN-205), AP018035 (HN-321) and AP018036(HN-506)-2017.

## Supplementary Data

Supplementary File 1
