# Supplementary material for: A complete high-quality MinION nanopore assembly of an extensively drug-resistant Mycobacterium tuberculosis Beijing lineage strain identifies novel variation in repetitive PE/PPE gene regions
Source: Microb Genom. 2018 Jun 15;4(7):e000188. doi: 10.1099/mgen.0.000188 (PMC6113869; doi:10.1099/mgen.0.000188)
Supplement: Supplementary File 1 [file mgen-4-188-s001.pdf]

**Supplementary material**

**Fig. S1**: A scheme of workflow to generate variants from Oxford Nanopore technologies (ONT) and Illumina reads

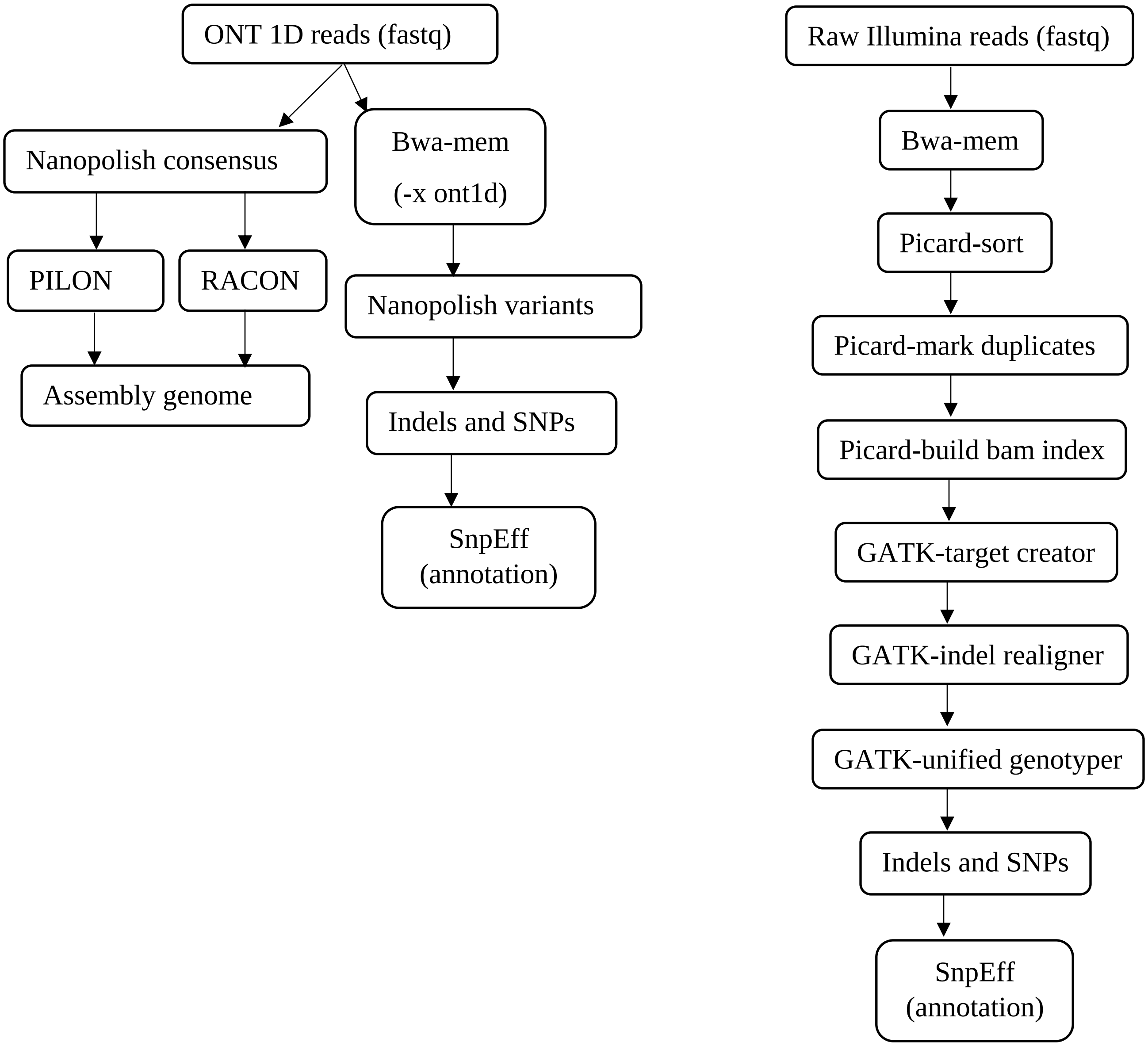

**Fig. S2**: Dot plot of sequence accuracy between the draft WP-XDR genome and the reference genome H37Rv which revealed 99.7% Average Nucleotide Identity (ANI) score. The red dots show a forward orientation while blue dots show reverse orientation

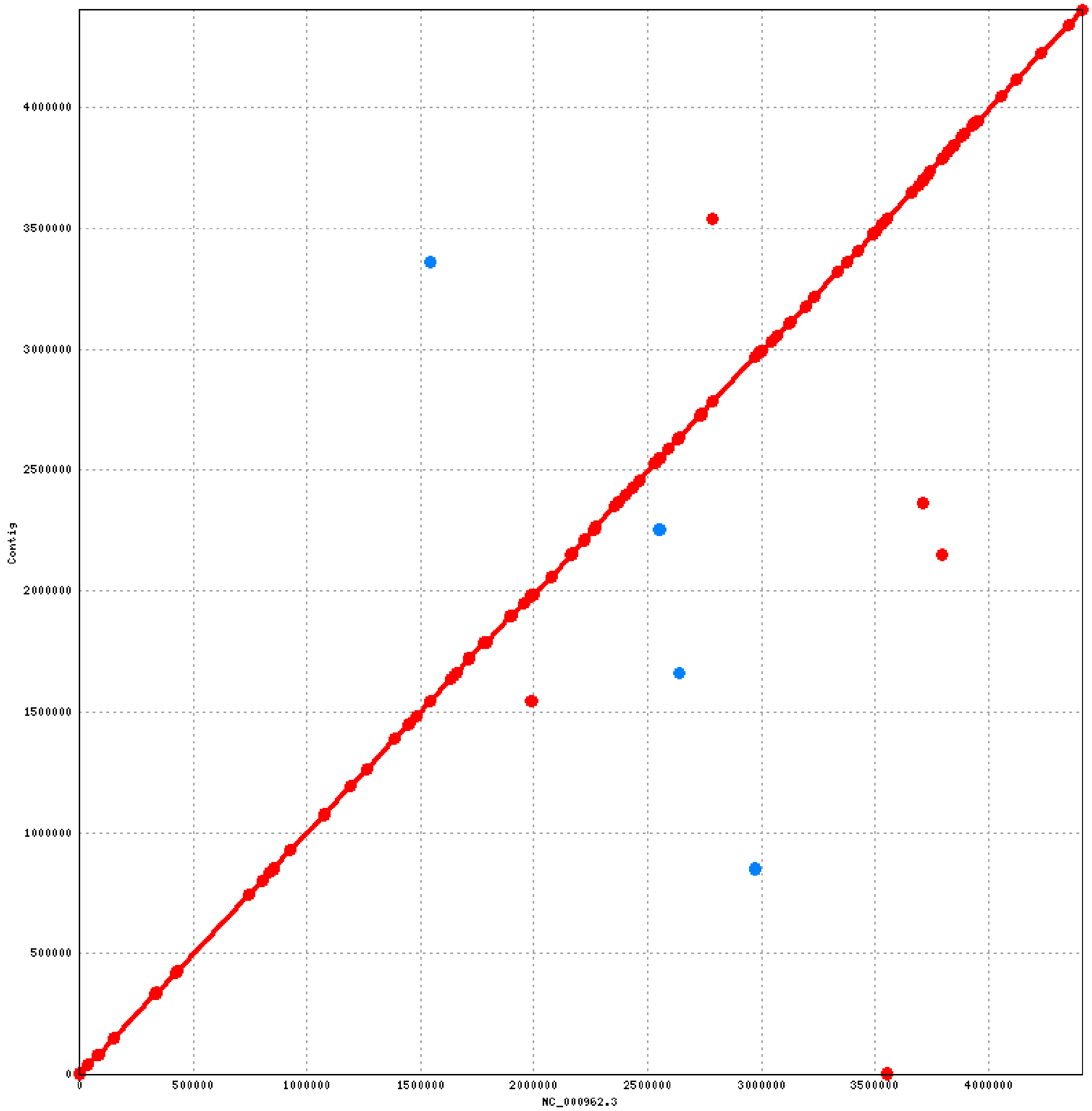

**Fig. S3**: Plot of sequence similarity of 168 PE/PPE family genes identified from the assembly and their sequence and depth coverage from Oxford minion® reads. The marks on the x-axis represent the different PE/PPE genes while y-axis respects their percentage sequence similarity from blastn (red), percentage breadth of coverage (black), and read depth (blue). Sample was sequenced to an average depth of 238X

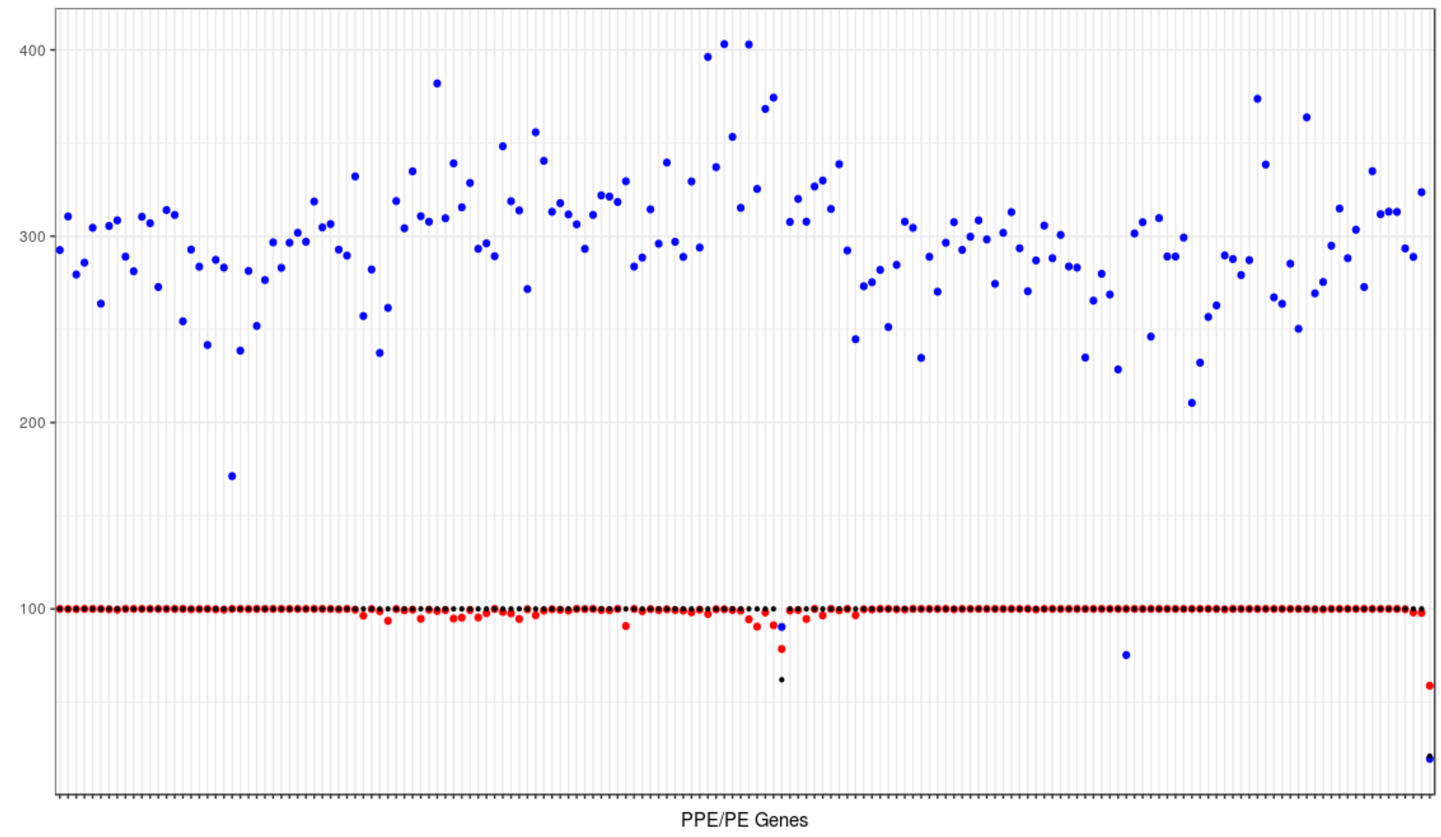

**Fig. S4**: Plot of sequence and depth coverage for the assembly of 168 PE/PPE family genes using Illumina reads. The marks on the x-axis represent the different 168 PE/PPE family genes while y-axis respective their percentage breadth of coverage (red), and read depth (blue). Sample was sequenced to average 46.3x depth

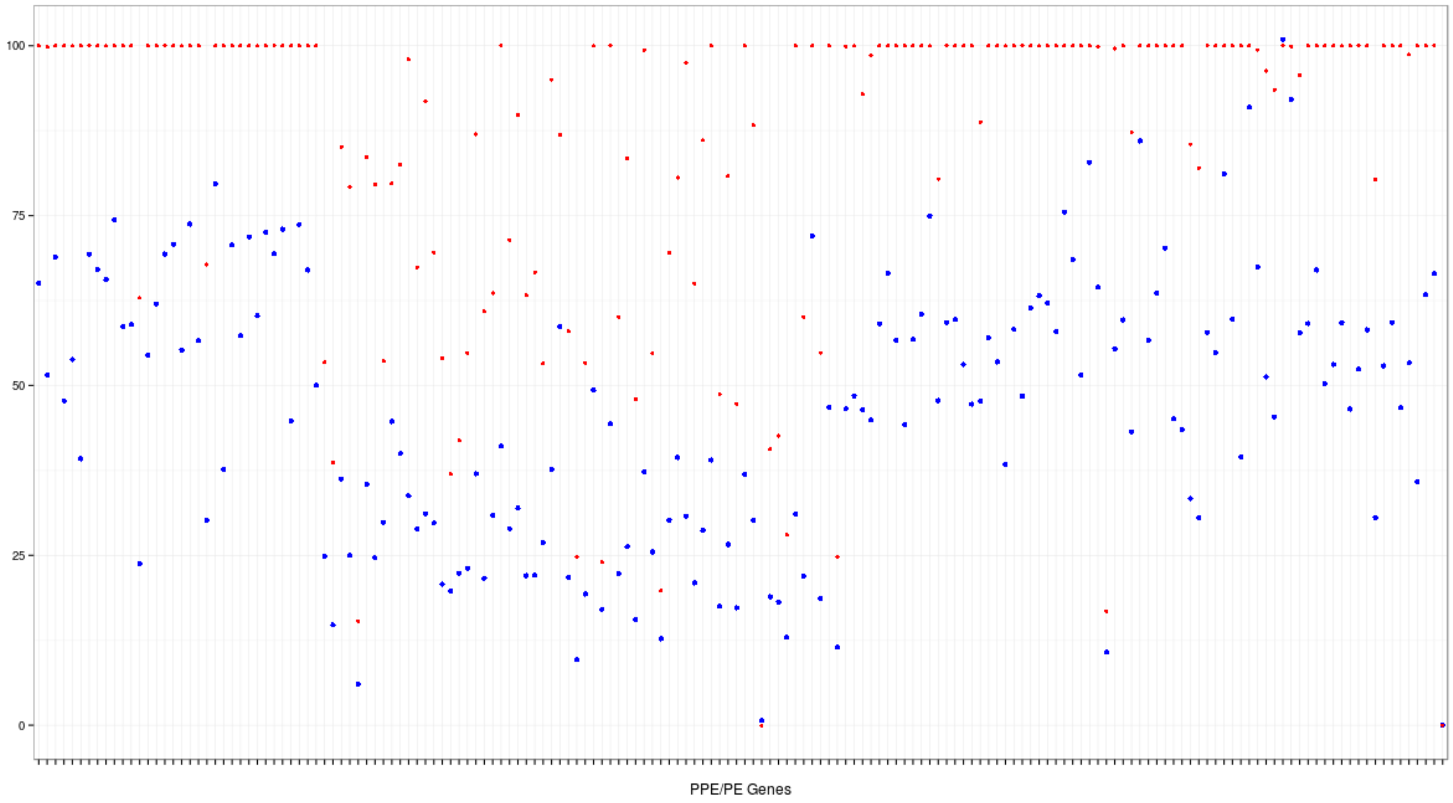

**Fig. S5**: Protein structure of the PPE family protein predicted using Phyre2 for a 654bp gene sequence with an end 323bp insertion identified within the assembled genome but absent in the reference genome H37Rv.

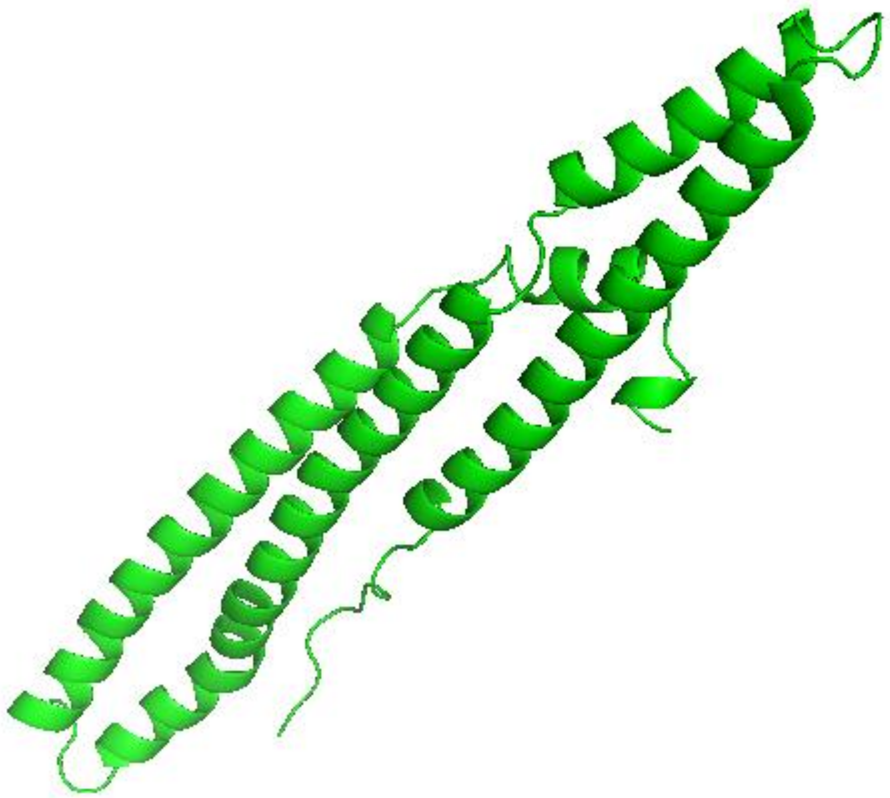

**Table S1:** List of PE/PPE family genes and their genomic position in H37Rv (NC_000962.3)

| Locus Number | Gene | Start | End |
|---|---|---|---|
| Rv0096 | PPE1 | 105324 | 106715 |
| Rv0109 | PE_PGRS1 | 131382 | 132872 |
| Rv0124 | PE_PGRS2 | 149533 | 150996 |
| Rv0151c | PE1 | 177543 | 179309 |
| Rv0152c | PE2 | 179319 | 180896 |
| Rv0159c | PE3 | 187433 | 188849 |
| Rv0160c | PE4 | 188931 | 190451 |
| Rv0256c | PPE2 | 307877 | 309547 |
| Rv0278c | PE_PGRS3 | 333437 | 336310 |
| Rv0279c | PE_PGRS4 | 336560 | 339073 |
| Rv0280 | PPE3 | 339364 | 340974 |
| Rv0285 | PE5 | 349624 | 349932 |
| Rv0286 | PPE4 | 349935 | 351476 |
| Rv0297 | PE_PGRS5 | 361334 | 363109 |
| Rv0304c | PPE5 | 366150 | 372764 |
| Rv0305c | PPE6 | 372820 | 375711 |
| Rv0335c | PE6 | 399535 | 400050 |
| Rv0354c | PPE7 | 424269 | 424694 |
| Rv0355c | PPE8 | 424777 | 434679 |
| Rv0388c | PPE9 | 467459 | 468001 |
| Rv0442c | PPE10 | 530751 | 532214 |
| Rv0453 | PPE11 | 543174 | 544730 |
| Rv0532 | PE_PGRS6 | 622793 | 624577 |
| Rv0578c | PE_PGRS7 | 671996 | 675916 |
| Rv0742 | PE_PGRS8 | 832981 | 833508 |
| Rv0746 | PE_PGRS9 | 835701 | 838052 |
| Rv0747 | PE_PGRS10 | 838451 | 840856 |
| Rv0754 | PE_PGRS11 | 846159 | 847913 |
| Rv0755c | PPE12 | 848103 | 850040 |
| Rv0832 | PE_PGRS12 | 924951 | 925364 |
| Rv0833 | PE_PGRS13 | 925361 | 927610 |
| Rv0834c | PE_PGRS14 | 927837 | 930485 |
| Rv0872c | PE_PGRS15 | 968424 | 970244 |
| Rv0878c | PPE13 | 976872 | 978203 |
| Rv0915c | PPE14 | 1020058 | 1021329 |
| Rv0916c | PE7 | 1021344 | 1021643 |
| Rv0977 | PE_PGRS16 | 1090373 | 1093144 |
| Rv0978c | PE_PGRS17 | 1093361 | 1094356 |

| | | | |
|---|---|---|---|
| Rv0980c | PE_PGRS18 | 1095078 | 1096451 |
| Rv1039c | PPE15 | 1161297 | 1162472 |
| Rv1040c | PE8 | 1162549 | 1163376 |
| Rv1067c | PE_PGRS19 | 1188421 | 1190424 |
| Rv1068c | PE_PGRS20 | 1190757 | 1192148 |
| Rv1087 | PE_PGRS21 | 1211560 | 1213863 |
| Rv1088 | PE9 | 1214513 | 1214947 |
| Rv1089 | PE10 | 1214769 | 1215131 |
| Rv1091 | PE_PGRS22 | 1216469 | 1219030 |
| Rv1135c | PPE16 | 1262272 | 1264128 |
| Rv1168c | PPE17 | 1298764 | 1299804 |
| Rv1169c | lipX | 1299822 | 1300124 |
| Rv1172c | PE12 | 1301755 | 1302681 |
| Rv1195 | PE13 | 1339003 | 1339302 |
| Rv1196 | PPE18 | 1339349 | 1340524 |
| Rv1214c | PE14 | 1357293 | 1357625 |
| Rv1243c | PE_PGRS23 | 1384989 | 1386677 |
| Rv1325c | PE_PGRS24 | 1488154 | 1489965 |
| Rv1361c | PPE19 | 1532443 | 1533633 |
| Rv1386 | PE15 | 1561464 | 1561772 |
| Rv1387 | PPE20 | 1561769 | 1563388 |
| Rv1396c | PE_PGRS25 | 1572127 | 1573857 |
| Rv1430 | PE16 | 1606386 | 1607972 |
| Rv1441c | PE_PGRS26 | 1618209 | 1619684 |
| Rv1450c | PE_PGRS27 | 1630638 | 1634627 |
| Rv1452c | PE_PGRS28 | 1636004 | 1638229 |
| Rv1468c | PE_PGRS29 | 1655609 | 1656721 |
| Rv1548c | PPE21 | 1751297 | 1753333 |
| Rv1646 | PE17 | 1855764 | 1856696 |
| Rv1651c | PE_PGRS30 | 1862347 | 1865382 |
| Rv1705c | PPE22 | 1931497 | 1932654 |
| Rv1706c | PPE23 | 1932694 | 1933878 |
| Rv1753c | PPE24 | 1981614 | 1984775 |
| Rv1759c | wag22 | 1989833 | 1992577 |
| Rv1768 | PE_PGRS31 | 2000614 | 2002470 |
| Rv1787 | PPE25 | 2025301 | 2026398 |
| Rv1788 | PE18 | 2026477 | 2026776 |
| Rv1789 | PPE26 | 2026790 | 2027971 |
| Rv1790 | PPE27 | 2028425 | 2029477 |
| Rv1791 | PE19 | 2029904 | 2030203 |
| Rv1800 | PPE28 | 2039453 | 2041420 |
| Rv1801 | PPE29 | 2042001 | 2043272 |
| Rv1802 | PPE30 | 2043384 | 2044775 |

| | | | |
|---|---|---|---|
| Rv1803c | PE_PGRS32 | 2044923 | 2046842 |
| Rv1806 | PE20 | 2048072 | 2048371 |
| Rv1807 | PPE31 | 2048398 | 2049597 |
| Rv1808 | PPE32 | 2049921 | 2051150 |
| Rv1809 | PPE33 | 2051282 | 2052688 |
| Rv1818c | PE_PGRS33 | 2061178 | 2062674 |
| Rv1840c | PE_PGRS34 | 2087971 | 2089518 |
| Rv1917c | PPE34 | 2162932 | 2167311 |
| Rv1918c | PPE35 | 2167649 | 2170612 |
| Rv1983 | PE_PGRS35 | 2226244 | 2227920 |
| Rv2098c | PE_PGRS36 | 2356729 | 2358033 |
| Rv2099c | PE21 | 2358033 | 2358206 |
| Rv2107 | PE22 | 2367359 | 2367655 |
| Rv2108 | PPE36 | 2367711 | 2368442 |
| Rv2123 | PPE37 | 2381071 | 2382492 |
| Rv2126c | PE_PGRS37 | 2387202 | 2387972 |
| Rv2162c | PE_PGRS38 | 2423240 | 2424838 |
| Rv2328 | PE23 | 2600731 | 2601879 |
| Rv2340c | PE_PGRS39 | 2617667 | 2618908 |
| Rv2352c | PPE38 | 2632923 | 2634098 |
| Rv2353c | PPE39 | 2634528 | 2635592 |
| Rv2356c | PPE40 | 2637688 | 2639535 |
| Rv2371 | PE_PGRS40 | 2651753 | 2651938 |
| Rv2396 | PE_PGRS41 | 2692799 | 2693884 |
| Rv2408 | PE24 | 2706017 | 2706736 |
| Rv2430c | PPE41 | 2727336 | 2727920 |
| Rv2431c | PE25 | 2727967 | 2728266 |
| Rv2487c | PE_PGRS42 | 2795301 | 2797385 |
| Rv2490c | PE_PGRS43 | 2801254 | 2806236 |
| Rv2519 | PE26 | 2835785 | 2837263 |
| Rv2591 | PE_PGRS44 | 2921551 | 2923182 |
| Rv2608 | PPE42 | 2935046 | 2936788 |
| Rv2615c | PE_PGRS45 | 2943600 | 2944985 |
| Rv2634c | PE_PGRS46 | 2960105 | 2962441 |
| Rv2741 | PE_PGRS47 | 3053914 | 3055491 |
| Rv2768c | PPE43 | 3076894 | 3078078 |
| Rv2769c | PE27 | 3078158 | 3078985 |
| Rv2770c | PPE44 | 3079309 | 3080457 |
| Rv2853 | PE_PGRS48 | 3162268 | 3164115 |
| Rv2892c | PPE45 | 3200794 | 3202020 |
| Rv3018c | PPE46 | 3376939 | 3378243 |
| Rv3018A | PE27A | 3378329 | 3378415 |
| Rv3021c | PPE47 | 3379376 | 3380452 |

| | | | |
|---|---|---|---|
| Rv3022c | PPE48 | 3380440 | 3380682 |
| Rv3022A | PE29 | 3380679 | 3380993 |
| Rv3097c | lipY | 3465778 | 3467091 |
| Rv3125c | PPE49 | 3490476 | 3491651 |
| Rv3135 | PPE50 | 3501334 | 3501732 |
| Rv3136 | PPE51 | 3501794 | 3502936 |
| Rv3144c | PPE52 | 3510088 | 3511317 |
| Rv3159c | PPE53 | 3527391 | 3529163 |
| Rv3343c | PPE54 | 3729364 | 3736935 |
| Rv3344c | PE_PGRS49 | 3736984 | 3738438 |
| Rv3345c | PE_PGRS50 | 3738158 | 3742774 |
| Rv3347c | PPE55 | 3743711 | 3753184 |
| Rv3350c | PPE56 | 3755952 | 3767102 |
| Rv3367 | PE_PGRS51 | 3778568 | 3780334 |
| Rv3388 | PE_PGRS52 | 3801653 | 3803848 |
| Rv3425 | PPE57 | 3842239 | 3842769 |
| Rv3426 | PPE58 | 3843036 | 3843734 |
| Rv3429 | PPE59 | 3847165 | 3847701 |
| Rv3477 | PE31 | 3894093 | 3894389 |
| Rv3478 | PPE60 | 3894426 | 3895607 |
| Rv3507 | PE_PGRS53 | 3926569 | 3930714 |
| Rv3508 | PE_PGRS54 | 3931005 | 3936710 |
| Rv3511 | PE_PGRS55 | 3939617 | 3941761 |
| Rv3512 | PE_PGRS56 | 3941724 | 3944963 |
| Rv3514 | PE_PGRS57 | 3945794 | 3950263 |
| Rv3532 | PPE61 | 3969343 | 3970563 |
| Rv3533c | PPE62 | 3970705 | 3972453 |
| Rv3539 | PPE63 | 3978059 | 3979498 |
| Rv3558 | PPE64 | 3997980 | 3999638 |
| Rv3590c | PE_PGRS58 | 4031404 | 4033158 |
| Rv3595c | PE_PGRS59 | 4036731 | 4038050 |
| Rv3621c | PPE65 | 4060648 | 4061889 |
| Rv3622c | PE32 | 4061899 | 4062198 |
| Rv3650 | PE33 | 4091233 | 4091517 |
| Rv3652 | PE_PGRS60 | 4093632 | 4093946 |
| Rv3653 | PE_PGRS61 | 4093940 | 4094527 |
| Rv3738c | PPE66 | 4189285 | 4190232 |
| Rv3739c | PPE67 | 4190284 | 4190517 |
| Rv3746c | PE34 | 4196171 | 4196506 |
| Rv3812 | PE_PGRS62 | 4276571 | 4278085 |
| Rv3872 | PE35 | 4350745 | 4351044 |
| Rv3873 | PPE68 | 4351075 | 4352181 |
| Rv3892c | PPE69 | 4374484 | 4375683 |

| | | | |
|---|---|---|---|
| Rv3893c | PE36 | 4375762 | 4375995 |

**Table S2:** Non-consensus SNPs identified from ONT reads mapped to reference H37Rv (159/1254 SNPs)

| SNP | COUNT | Proportion (%) |
|---|---|---|
| A->C | 6 | 0.48 |
| A->G | 15 | 1.20 |
| T->C | 18 | 1.44 |
| T-G | 13 | 1.04 |
| C->A | 9 | 0.72 |
| C->T | 19 | 1.52 |
| C->G | 23 | 1.83 |
| G->A | 22 | 1.75 |
| G->T | 20 | 1.59 |
| G->C | 14 | 1.12 |

**Table S3**: Number of SNPs and average base coverage of PE/PPE family genes identified from ONT and Illumina reads

| PE/PPE genes | ONT | Illumina | Overlap SNPs | Illumina average base coverage | ONT average base coverage |
|---|---|---|---|---|---|
| PPE1 | 1 | 1 | 1 | 46 | 273 |
| PE_PGRS1 | 1 | 0 | 0 | 24 | 332 |
| PE1 | 1 | 1 | 1 | 65 | 279 |
| PE3 | 1 | 1 | 1 | 79 | 281 |
| PPE2 | 2 | 2 | 2 | 47 | 307 |
| PPE3 | 1 | 2 | 2 | 61 | 305 |
| PPE6 | 2 | 2 | 2 | 59 | 269 |
| PPE47 | 0 | 14 | 0 | 19 | 232 |
| PPE8 | 4 | 4 | 4 | 53 | 288 |
| PE_PGRS6 | 3 | 0 | 0 | 12 | 307 |
| PE_PGRS7 | 4 | 2 | 2 | 18 | 338 |
| PPE9 | 0 | 9 | 0 | 35 | 323 |

| | | | | | |
|---|---|---|---|---|---|
| PE_PGRS9 | 2 | 0 | 0 | 11 | 244 |
| PE_PGRS10 | 6 | 4 | 4 | 14 | 257 |
| PE_PGRS13 | 3 | 0 | 0 | 6 | 261 |
| PE_PGRS14 | 1 | 0 | 0 | 35 | 318 |
| PE_PGRS15 | 1 | 0 | 0 | 24 | 304 |
| PE_PGRS17 | 3 | 1 | 1 | 44 | 310 |
| PE_PGRS18 | 4 | 0 | 0 | 39 | 307 |
| PPE16 | 0 | 3 | 0 | 44 | 234 |
| PE8 | 1 | 1 | 1 | 66 | 292 |
| PE_PGRS20 | 4 | 0 | 0 | 31 | 339 |
| PE_PGRS44 | 0 | 2 | 0 | 37 | 339 |
| PE_PGRS21 | 2 | 0 | 0 | 29 | 315 |
| PE_PGRS22 | 5 | 2 | 2 | 20 | 328 |
| PPE17 | 1 | 1 | 1 | 56 | 288 |
| PPE18 | 1 | 0 | 0 | 60 | 270 |
| PE14 | 1 | 1 | 1 | 53 | 305 |
| PPE19 | 2 | 0 | 0 | 74 | 296 |
| PPE20 | 1 | 1 | 1 | 59 | 292 |
| PPE31 | 0 | 1 | 0 | 62 | 313 |
| PE_PGRS25 | 1 | 1 | 1 | 23 | 289 |
| PE16 | 1 | 1 | 1 | 69 | 289 |
| PE_PGRS26 | 1 | 1 | 1 | 37 | 348 |
| PE_PGRS27 | 2 | 0 | 0 | 21 | 318 |
| PE_PGRS28 | 6 | 3 | 3 | 30 | 313 |
| PPE21 | 1 | 0 | 0 | 59 | 299 |
| PPE22 | 1 | 1 | 1 | 53 | 308 |
| PPE24 | 2 | 0 | 0 | 47 | 274 |
| PPE30 | 1 | 0 | 0 | 63 | 288 |
| PE_PGRS32 | 3 | 1 | 1 | 22 | 317 |
| PPE32 | 2 | 0 | 0 | 57 | 283 |
| PPE33 | 2 | 2 | 2 | 75 | 283 |
| PE_PGRS33 | 1 | 0 | 0 | 26 | 311 |
| PPE34 | 2 | 3 | 2 | 68 | 234 |
| PPE35 | 2 | 1 | 1 | 51 | 265 |
| PPE37 | 1 | 0 | 0 | 64 | 268 |
| PE_PGRS37 | 1 | 0 | 0 | 9 | 321 |
| PE23 | 2 | 2 | 2 | 62 | 292 |
| PE24 | 1 | 1 | 1 | 69 | 274 |
| PE_PGRS42 | 1 | 0 | 0 | 26 | 314 |
| PE_PGRS43 | 8 | 0 | 0 | 15 | 295 |
| PE26 | 1 | 1 | 1 | 55 | 287 |
| PE_PGRS47 | 4 | 0 | 0 | 30 | 329 |
| PE27 | 2 | 2 | 2 | 73 | 283 |

| | | | | | |
|---|---|---|---|---|---|
| PPE44 | 1 | 1 | 1 | 70 | 289 |
| PE_PGRS48 | 3 | 1 | 1 | 39 | 293 |
| lipY | 2 | 1 | 1 | 66 | 310 |
| PPE51 | 1 | 0 | 0 | 59 | 279 |
| PPE52 | 1 | 0 | 0 | 39 | 287 |
| PPE53 | 1 | 0 | 0 | 90 | 373 |
| PPE54 | 11 | 9 | 9 | 67 | 338 |
| PE_PGRS50 | 1 | 1 | 1 | 28 | 403 |
| PPE55 | 7 | 17 | 7 | 51 | 267 |
| PPE56 | 3 | 3 | 3 | 45 | 263 |
| PPE60 | 2 | 2 | 2 | 66 | 275 |
| PE_PGRS53 | 1 | 0 | 0 | 26 | 402 |
| PE_PGRS54 | 7 | 4 | 4 | 17 | 325 |
| PE_PGRS55 | 1 | 1 | 1 | 36 | 368 |
| PE_PGRS56 | 3 | 6 | 3 | 30 | 374 |
| PE_PGRS58 | 2 | 1 | 1 | 18 | 307 |
| PE_PGRS59 | 1 | 0 | 0 | 18 | 320 |
| PPE66 | 1 | 1 | 1 | 58 | 334 |
| PE35 | 1 | 0 | 0 | 60 | 296 |
| PPE69 | 1 | 1 | 1 | 59 | 313 |
| **Total** | **158** | **124** | **81** | | |

**Table S4:** Mutations identified in genes that encode for potential virulence proteins in theWP-XDR strain

| Gene | Nucleotide change | Position | Amino acid |
|---|---|---|---|
| *mak* | T->C | 154283 | p.Ser18Pro |
| *mce1A* | T->G | 199470 | p.Ser313Ala |
| *mce1D* | T->C | 203038 | p.Ile188Thr |
| *mce1F* | T->C | 206339 | p.Leu370Pro |
| *mce2A* | T->C | 686972 | p.Phe51Ser |
| *mce2F* | A->G | 694531 | p.Asn432Ser |
| *Rv0634c* | A->G | 731015 | p.Tyr7His |
| *mazF3* | G->A | 1230778 | p.Thr65Ile |
| *ephB* | G->T | 2191498 | p.Gly158Trp |
| *mce3A* | G->A | 2209465 | p.Ala47Thr |
| *mce3F* | C->A | 2216443 | p.Ala396Glu |
| *cstA* | C->A | 3428917 | p.Arg559Ser |
| *mce4C* | G->T | 3916386 | pArg191Ser |
| *proV* | T->C | 4204168 | p.Asn84Asp |
| *proX* | A->G | 4205120 | p.Leu85Pro |
| *mycP1* | T->C | 4364046 | Thr238Ala |

**Table S5**: Mutations in genes involved in cell wall biosynthesis identified in the WP-XDR strain

| Gene | Position | Mutation |
|---|---|---|
| *mmpL5* | 776182 | p.Asp767Asn |
| *mmpL12* | 1715165 | p.Ile816Met |
| *mmpL10* | 1322741 | p.Thr408Ala |
| *fadD6* | 1349733 | p.Glu134Asp |
| *fadE9* | 844023 | p.Asp131Gly |
| *fadD28* | 3284640 | p.Thr436Ala |
| *fadD30* | 484596 | p.Pro207Leu |
| *fadD34* | 37305 | p.Ser16Trp |
| *fadD34* | 37334 | p.Thr26Ser |
| *fadD36* | 1336164 | p.Pro124Leu |
| *fadD31* | 2177366 | p.Arg94Cys |
| *fadE17* | 2185674 | p.Ile172Thr |
| *fadE20* | 3036826 | p.Val156Met |
| *fadD28* | 3284640 | p.Thr436Ala |
| *fadE24* | 3505648 | p.Arg96Gly |
| *fadE26* | 3922552 | p.Leu218Met |
| *fadD19* | 3952308 | p.Asp55Tyr |
| *fadE35* | 4252066 | p.Ser328Pro |
| *fadD32* | 4262388 | p.Gly227Ser |
| *fadD23* | 4301075 | p.Glu422Gln |
| *pks5* | 1722228 | p.Leu2061Arg |
| *pks7* | 1876976 | p.Thr558Lys |
| *pks8* | 1885385 | p.Leu1228Val |
| *pks11* | 1891388 | p.Pro55Ala |
| *pks12* | 2295685 | p.Val3768Leu |
| *pks12* | 2302033 | p.Arg1652Cys |
| *pks13* | 4256210 | p.Ala1646Asp |

**Table S6**: Blast search results of the gene sequence with insertion confirming the uniqueness of insertion sequence among lineage 2 genomes

| Query Number | Reference genomes | Identification (%) | Identified (bp) | Query coverage (%) | start | end | E-value | Max score |
|---|---|---|---|---|---|---|---|---|
| 117001 | CP007027.1 | 100 | 332 | 50 | 3500716 | 3501047 | 1.11E-175 | 614 |
| 117001 | CP009480.1 | 100 | 332 | 50 | 3494479 | 3494810 | 1.11E-175 | 614 |
| 117001 | CP003248.2 | 100 | 332 | 50 | 3501331 | 3501662 | 1.11E-175 | 614 |
| 117001 | AL123456.3 | 100 | 332 | 50 | 3501334 | 3501665 | 1.11E-175 | 614 |
| 159769 | CP022014.1 | 100 | 654 | 100 | 3502186 | 3502839 | 0.00 | 654 |
| 159769 | AP018036.1 | 100 | 654 | 100 | 3489334 | 3489987 | 0.00 | 654 |
| 159769 | AP018035.1 | 100 | 654 | 100 | 3498189 | 3498842 | 0.00 | 654 |
| 159769 | AP018034.1 | 100 | 654 | 100 | 3486855 | 3487508 | 0.00 | 654 |
| 159769 | CP017920.1 | 100 | 654 | 100 | 4292533 | 4291880 | 0.00 | 654 |
| 159769 | CP013475.1 | 100 | 654 | 100 | 3481453 | 3482106 | 0.00 | 654 |
| 159769 | CP011510.1 | 100 | 654 | 100 | 4254434 | 4253781 | 0.00 | 654 |
| 159769 | CP016888.1 | 100 | 654 | 100 | 3496444 | 3497097 | 0.00 | 654 |
| 159769 | CP012506.2 | 100 | 654 | 100 | 3493237 | 3493890 | 0.00 | 654 |
| 159769 | CP016794.1 | 100 | 654 | 100 | 3493988 | 3494641 | 0.00 | 654 |
| 159769 | CP010968.1 | 100 | 654 | 100 | 3981473 | 3982126 | 0.00 | 654 |
| 159769 | CP010339.1 | 100 | 654 | 100 | 3499781 | 3500434 | 0.00 | 654 |
| 159769 | CP010335.1 | 100 | 654 | 100 | 3493892 | 3494545 | 0.00 | 654 |
| 159769 | CP012090.1 | 100 | 654 | 100 | 917081 | 916428 | 0.00 | 654 |
| 159769 | CP010895.1 | 100 | 654 | 100 | 3955706 | 3956359 | 0.00 | 654 |
| 159769 | CP007299.1 | 100 | 654 | 100 | 3490224 | 3490877 | 0.00 | 654 |
| 159769 | HG813240.1 | 100 | 654 | 100 | 3491900 | 3492553 | 0.00 | 654 |
| 159769 | CP009426.1 | 100 | 654 | 100 | 3470402 | 3471055 | 0.00 | 654 |
| 159769 | CP008983.1 | 100 | 654 | 100 | 518032 | 517379 | 0.00 | 654 |
| 159769 | CP008981.1 | 100 | 654 | 100 | 518033 | 517380 | 0.00 | 654 |
| 159769 | CP008980.1 | 100 | 654 | 100 | 518033 | 517380 | 0.00 | 654 |
| 159769 | CP008979.1 | 100 | 654 | 100 | 518032 | 517379 | 0.00 | 654 |
| 159769 | CP008978.1 | 100 | 654 | 100 | 518033 | 517380 | 0.00 | 654 |
| 159769 | CP008977.1 | 100 | 654 | 100 | 518032 | 517379 | 0.00 | 654 |
| 159769 | CP008976.1 | 100 | 654 | 100 | 518032 | 517379 | 0.00 | 654 |
| 159769 | CP008975.1 | 100 | 654 | 100 | 518032 | 517379 | 0.00 | 654 |
| 159769 | CP008974.1 | 100 | 654 | 100 | 518032 | 517379 | 0.00 | 654 |
| 159769 | CP008973.1 | 100 | 654 | 100 | 518033 | 517380 | 0.00 | 654 |
| 159769 | CP008972.1 | 100 | 654 | 100 | 518033 | 517380 | 0.00 | 654 |
| 159769 | CP008970.1 | 100 | 654 | 100 | 518032 | 517379 | 0.00 | 654 |

| | | | | | | | | |
|---|---|---|---|---|---|---|---|---|
| 159769 | CP008969.1 | 100 | 654 | 100 | 518033 | 517380 | 0.00 | 654 |
| 159769 | CP008968.1 | 100 | 654 | 100 | 518032 | 517379 | 0.00 | 654 |
| 159769 | CP008966.1 | 100 | 654 | 100 | 518032 | 517379 | 0.00 | 654 |
| 159769 | CP008964.1 | 100 | 654 | 100 | 518032 | 517379 | 0.00 | 654 |
| 159769 | CP008962.1 | 100 | 654 | 100 | 518033 | 517380 | 0.00 | 654 |
| 159769 | CP008961.1 | 100 | 654 | 100 | 518032 | 517379 | 0.00 | 654 |
| 159769 | CP008960.1 | 100 | 654 | 100 | 518033 | 517380 | 0.00 | 654 |
| 159769 | CP008959.1 | 100 | 654 | 100 | 518033 | 517380 | 0.00 | 654 |
| 159769 | CP007809.1 | 100 | 654 | 100 | 3488153 | 3488806 | 0.00 | 654 |
| 159769 | CP007803.1 | 100 | 654 | 100 | 3478240 | 3478893 | 0.00 | 654 |
| 159769 | CP002885.1 | 100 | 654 | 100 | 3491717 | 3492370 | 0.00 | 654 |
| 159769 | CP002883.1 | 100 | 654 | 100 | 3486486 | 3487139 | 0.00 | 654 |
| 159769 | CP002882.1 | 100 | 654 | 100 | 3480758 | 3481411 | 0.00 | 654 |
| 159769 | CP002871.1 | 100 | 654 | 100 | 3490732 | 3491385 | 0.00 | 654 |
| 159769 | CP002884.1 | 100 | 654 | 100 | 3492943 | 3493596 | 0.00 | 654 |
| 159769 | CP001642.1 | 100 | 654 | 100 | 3486529 | 3487182 | 0.00 | 654 |
| 159769 | CP001641.1 | 100 | 654 | 100 | 3478627 | 3479280 | 0.00 | 654 |
| 159769 | CP017598.1 | 100 | 654 | 100 | 3495720 | 3496373 | 0.00 | 654 |
| 159769 | CP017597.1 | 100 | 654 | 100 | 3522988 | 3523641 | 0.00 | 654 |
| 159769 | CP017596.1 | 100 | 654 | 100 | 3493170 | 3493823 | 0.00 | 654 |
| 159769 | CP017595.1 | 100 | 654 | 100 | 3508711 | 3509364 | 0.00 | 654 |
| 159769 | CP017594.1 | 100 | 654 | 100 | 3519190 | 3519843 | 0.00 | 654 |
| 159769 | CP017593.1 | 100 | 654 | 100 | 3505356 | 3506009 | 0.00 | 654 |
| 159769 | CP010873.1 | 100 | 654 | 100 | 3488175 | 3488828 | 0.00 | 654 |
| 159769 | CP008963.1 | 100 | 654 | 100 | 518033 | 517379 | 0.00 | 654 |

45
